# Cruciferous vegetables consumption and the risk of ovarian cancer: a meta-analysis of observational studies

**DOI:** 10.1186/1746-1596-9-7

**Published:** 2014-01-20

**Authors:** Bo Han, Xuepeng Li, Tao Yu

**Affiliations:** 1 Department of Obstetrics and Gynecology, Zaozhuang Municipal Hospital, Zaozhuang, Shandong, PR China

**Keywords:** Cruciferous vegetables, Ovarian cancer, Dietary, Risk factor, Meta-analysis

## Abstract

**Background:**

To quantify the effect of cruciferous vegetable consumption on the incidence of ovarian cancer by meta-analyzing the existing observational studies and provides quantitative and high-level evidence.

**Methods:**

A detailed literature search of Medline and EMBASE for all relevant papers published. A meta-analysis was conducted for the association between cruciferous vegetable consumption and risk of ovarian cancer.

**Results:**

A total of 4,306 cases in 375,562 controls in 11 independent studies were identified in this current meta-analysis. The result of this current meta-analysis, including 6 case-control and 5 cohort studies, indicated that cruciferous vegetable intake was associated with a reduced risk of ovarian cancer. Cruciferous vegetable consumption was associated with a reduced risk of ovarian cancer in case-control studies (RR=0.84; 95% CI, 0.75-0.94) but not in cohort studies (RR=1.00; 95% CI, 0.85-1.11).

**Conclusions:**

The results from this meta-analysis of observational studies demonstrate that cruciferous vegetable consumption is a prospective factor of the ovarian cancer. However, more in-depth studies are warranted to report more detailed results, including other specific vegetables within the cruciferous vegetable family.

**Virtual slides:**

The virtual slide (s) for this article can be found here: http://www.diagnosticpathology.diagnomx.eu/vs/1116708293115581.

## Introduction

It was reported that ovarian cancer is the eighth most common cancer and the fifth most common cause of cancer death in women in the developed countries [1]. Usually it is diagnosed at an advanced stage and, therefore, despite improvements in treatment, the survival rate remains low at less than 45% after 5 years [1-4]. Marked geographic variation in incidence rates suggests an important role of behavioral and potentially modifiable factors such as diet in ovarian cancer development. However, there is no clear etiologic role of dietary intake in ovarian cancer [5]. Some epidemiological studies have shown that high consumption of vegetables reduces the risk of ovarian cancer; however, daily intake of red meat was significantly associated with the risk of ovarian cancer [6,7]. It is important to detect the harmful or protective factors for the ovarian cancer. The realization of the relationship between the modifiable epidemiological factors and risk of ovarian cancer would provide a more effective strategy for the cancer prevention in the future.

Cruciferous vegetables are a special group of vegetables named for their cross-shaped flower petals, including cabbage, broccoli, brussels sprouts, cauliflower and other members of the family. There is accumulating evidence that cruciferous vegetable consumption may lower the risk for several types of cancers [8]. Although several epidemiological studies have focused on the association between cruciferous vegetable intake and ovarian cancer risk, their conclusions have been inconsistent. Meta-analysis is a useful statistical tool to pool the relevant studies together and gain a more powerful conclusion [9,10]. The meta-analysis was also used in the search for potential causes of ovarian cancer. We therefore conducted a meta-analysis of all published studies to gain a better understanding of the relationship between cruciferous vegetables and ovarian cancer risk.

## Methods

### Search strategy and inclusion criteria

We followed the Meta-Analysis of Observational Studies in Epidemiology (MOOSE) [11] and Preferred reporting items for systematic reviews and meta-analyses (PRISMA) [12] guidelines in conducting this meta-analysis. A systematic literature search was conducted through two electronic databases (Medline and EMBASE) until Dec. 5, 2013. The key words “cruciferous vegetable*” or “brassica”, “diet” and “ovarian cancer” were searched as text word and exploded as medical subject headings (MeSH) where possible. The reference lists of relevant articles were reviewed for the additional studies. No language or other restrictions were set in the literature search or the inclusion criteria. If additional data was required, the corresponding authors will be contacted.

The studies were be considered included if they met the following inclusion criteria: 1) studies reported the association between cruciferous vegetable intake and risk of ovarian cancer; 2) studies obtained a case–control or cohort study design; 3) the value of relative risk (RR), odds ratio (OR) with 95% confidence intervals (CI) or the raw data to calculate them were reported.

### Data extraction and assessment of study quality

The data extraction was conducted via a standardized data extraction form, collecting information on the name of first author, the publication year, study design, number of cases and controls, sample size, study site, adjustments of the confounding factors, and the OR/RR value with 95% CI. When the OR or RR was not reported in the article, the RR with 95% CI with the raw data and no confounding factors were adjusted.

The study quality was assessed by two reviewers back to back and any discrepancies were re solved by reevaluating the included articles and discussion with a third investigator. We obtained the Newcastle-Ottawa Scale (NOS) Assessment of the quality of the included studies [13]. The study quality was assigned to each study based on the 3 parts: selection, comparability, and exposure and outcome condition. The NOS assessed the selection, comparability and exposure of a case–control study, while the selection, comparability and outcome of a cohort study. The study with more than 6 stars would be regarded in relative high quality.

### Data integration and statistical methods

The RR was obtained to approximate RR in this meta-analysis because of the low incidence rate of ovarian cancer. When both the crude and the adjusted OR/RR values were offered, only the adjusted value would be adopted for the meta-analysis. If only the raw data was reported, we would calculate the unadjusted RR.

The heterogeneity among the included studies was measured by the χ^2^ test and quantified with the I^2^ statistic. When *P* for the heterogeneity was < 0.1 and *I*^*2*^ > 50%, the interstudy heterogeneity would be considered statistically significant. The ORs and 95% CIs of all the included studies were pooled using the general variance-based method with a fixed-effects model unless the heterogeneity is significant. The source of the statistically significant heterogeneity was assessed by both removing the included studies one by one to measure whether any single study was the source of the heterogeneity. Another independent method to detect the source of heterogeneity was to conduct a subgroup meta-analysis. Subgroup analyses were conducted by the study designs (case–control or cohort study) and population of or hospital based design.

A sensitivity analysis was performed by excluding the included studies from the meta-analysis. The publication bias was evaluated using funnel plots and the Egger test [14,15]. P < 0.1 was considered to indicate statistically significant publication bias. All analyses were conducted using STATA software, version 12.0 (StataCorp LP, College Station, Texas).

## Results

### Identification and selection of studies

**Figure 1:**
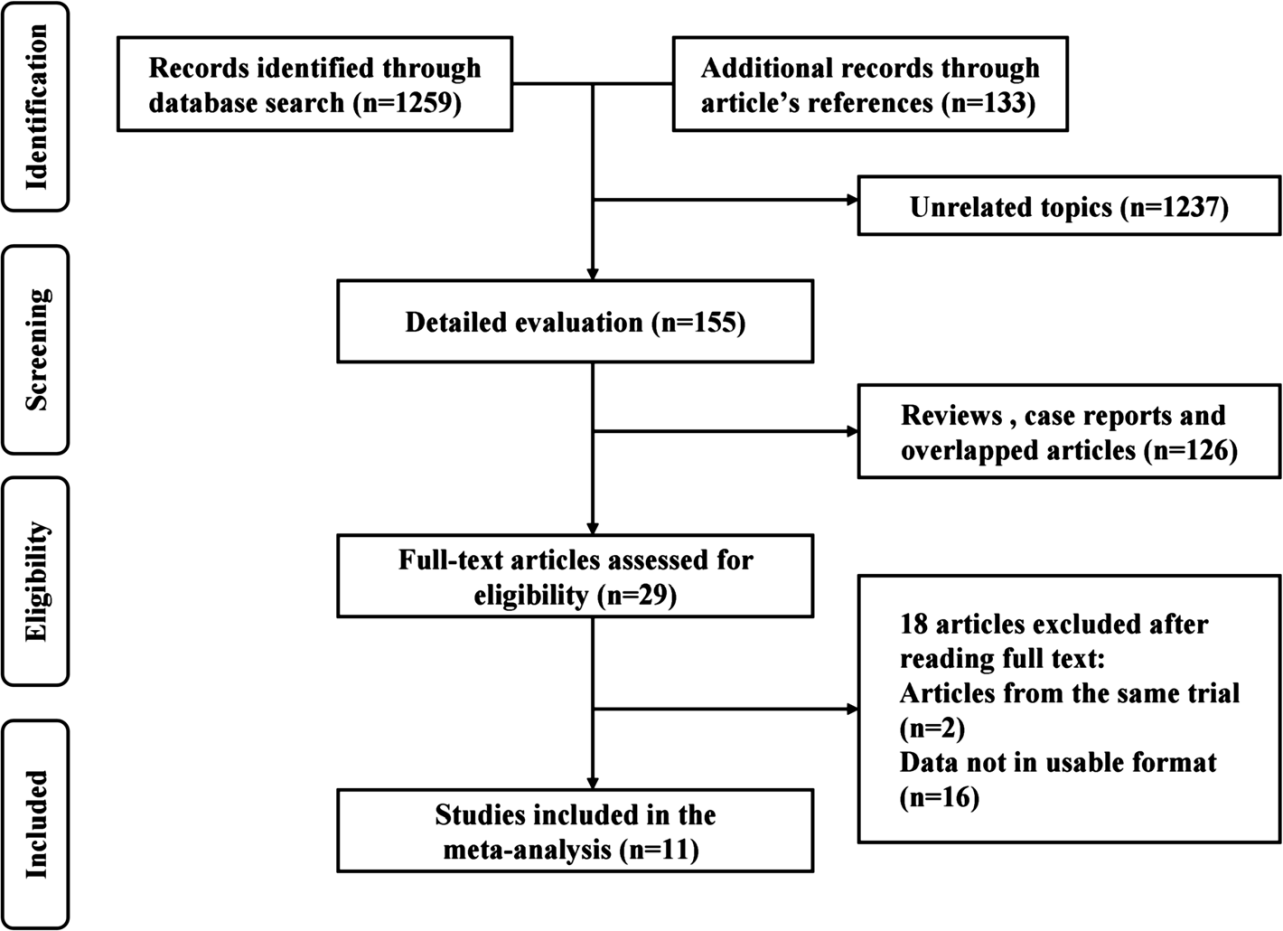
**Flow chart of the literature search.** The literature search was conducted in Medline and EMBASE. The reference lists of the relevant studies were reviewed as well.

The flowchart of the study selection was presented in Figure 1. A total of 1392 publications were retrieved from the initial literature search (524 form the Medline, 735 from the EMBASE, and 133 from the reference lists of the relevant studies). After excluding 1237 articles with unrelated topics, a total of 155 records were detailed evaluated. Among the 155 articles, 29 full-texts were assessed for eligibility after removing 126 articles (reviews, case reports and overlapped articles). From these, 11 original articles that included data on the association between cruciferous vegetables consumption and ovarian cancer were ultimately included in our meta-analysis [9,16-25].

### Study characteristics and quality

A total of 4,306 cases in 375,562 controls in 11 independent studies were identified. Among the 11 studies, there were 5 cohort studies and 6 case–control studies. Overall, 7 studies was a population based study and the rest 4 studies were hospital based studies. The geographicical distribution of the studies sties was 4 were in European, 4 in Americas, 2 in Asia and 1 in Australia. The detailed age, gender distribution, duration of studies, and adjustments of confounding factors were demonstrated in Table 1.

**Table 1:** Study characteristics of published cohort and case–control studies on egg intake and ovarian cancer

**Author publication year**	**Country**	**Study period**	**Study design**	**No. of subjects**	**Cases**	**FFQ used for dietary assessment**	**Confounders for adjustment**
La Vecchia C1987	Italy	1979-1986	Hospital-based case–control study	1,380	445	Yes	Age, interviewer, marital status, social class, education, parity, age at first birth, age at menarche, menopausal status, age at menopause, BMI, oral contraceptive and other female hormone use, retinol and carotene indices, added score of fat consumption and alcohol intake
Engle A 1991	USA	1984-1989	Hospital-based case–control study	212	72	Yes	Age and smoking status
Fairfield KM 2001	USA	1976-1996	Population-based cohort study	80,326	527	Yes	Age, BMI, duration of oral contraception use, smoking history, parity, history of tubal ligation, total energy, and dietary fiber
Zhang M 2002	China	1999–2000	Hospital-based case–control study	906	254	Yes	Age at interview, education, living area, BMI, smoking, alcohol drinking, tea drinking, family income, marital and menopause status, parity, tubal ligation, oral contraceptive use, physical activity, family history of ovarian cancer, total energy intake, fruit, milk, fish, meat, egg intake
Pan SY 2004	Canada	1994-1997	Case–control study population-based	2,577	442	Yes	Age, province of residence, education, alcohol consumption, cigarette pack-years, BMI, total caloric intake, recreational physical activity, number of live births, menstruation years, and menopause status
Larsson SC 2004	Sweden	1987–1990	Population-based cohort study	61,084	266	Yes	Age, BMI, educational level, parity, oral contraceptive use, fish consumption, and dietary lactose intake, consumption of total fruit
Mommers M 2005	Netherlands	1986-1997	Population-based cohort study	62,573	252	Yes	Age, height, current cigarette smoker, duration of cigarette smoking, number of cigarettes smoked daily, duration of oral contraceptive use, parity, total fruit intake; total vegetable intake; all individual fruit or vegetable items listed for all other individual fruit or vegetable item
Sakauchi F 2007	Japan	1988-2003	Population-based cohort study	64,327	54	Yes	Age, menopausal status, number of pregnancies, history of sex hormone use, BMI, physical activity, and education
Chang ET 2007	USA	1995-2003	Population-based cohort study	97,275	280	Yes	Age, race, total energy intake, parity, oral contraceptive use, strenuous exercise, wine consumption, and menopausal status/hormone therapy use
Kolahdooz F 2009	Australia	1990-1993	Population-based case–control study	1,460	683	Yes	Age, oral contraceptive use, parity, education after high school, and energy intake
Bosetti C 2012	Italy and Switzerland	1991-2009	Hospital-based case–control study	3,442	1,031	Yes	Age, study center, year of interview, education, BMI, alcohol drinking, tobacco smoking, and total energy intake

BMI = body mass index; NR = not reported; FFQ = food frequency questionnaire.

The methodological qualities of the included studies were conducted by the Newcastle-Ottawa Scale (NOS) Assessment.

Study quality was judged on the basis of the Newcastle-Ottawa Scale (1–9 stars). The scale distribution was from 5 to 8 stars. Among the 11 included studies, all studies demonstrated a relatively high quality (more than 6 stars in NOS) (Table 2).

**Table 2:** Subgroup analysis of cruciferous vegetables consumption and the risk of ovarian cancer

	**No. of studies**	**Pooled estimate**		**Tests of heterogeneity**	
		**RR**	**95% CI**	**P value**	**I**^**2 **^**(%)**
All studies	11	0.90	0.82- 0.98	0.204	25.2
Study design					
Cohort	5	1.00	0.85-1.11	0.897	0.0
Case–control	6	**0.84**	**0.75-0.94**	0.091	47.4
Study location					
Europe	4	**0.88**	**0.79-0.99**	0.656	0.0
North America	4	0.88	0.77-1.02	0.043	63.2
Asia	2	0.84	0.53-1.34	0.217	34.5
Australia	1	1.16	0.81-1.67	/	/
Data source					
Hospital-based	4	**0.82**	**0.72-0.94**	0.119	48.8
Population-based	7	0.95	0.85-1.07	0.577	0.0

The data in bold demonstrate significant results.

### Cruciferous vegetable consumption and ovarian cancer

**Figure 2:**
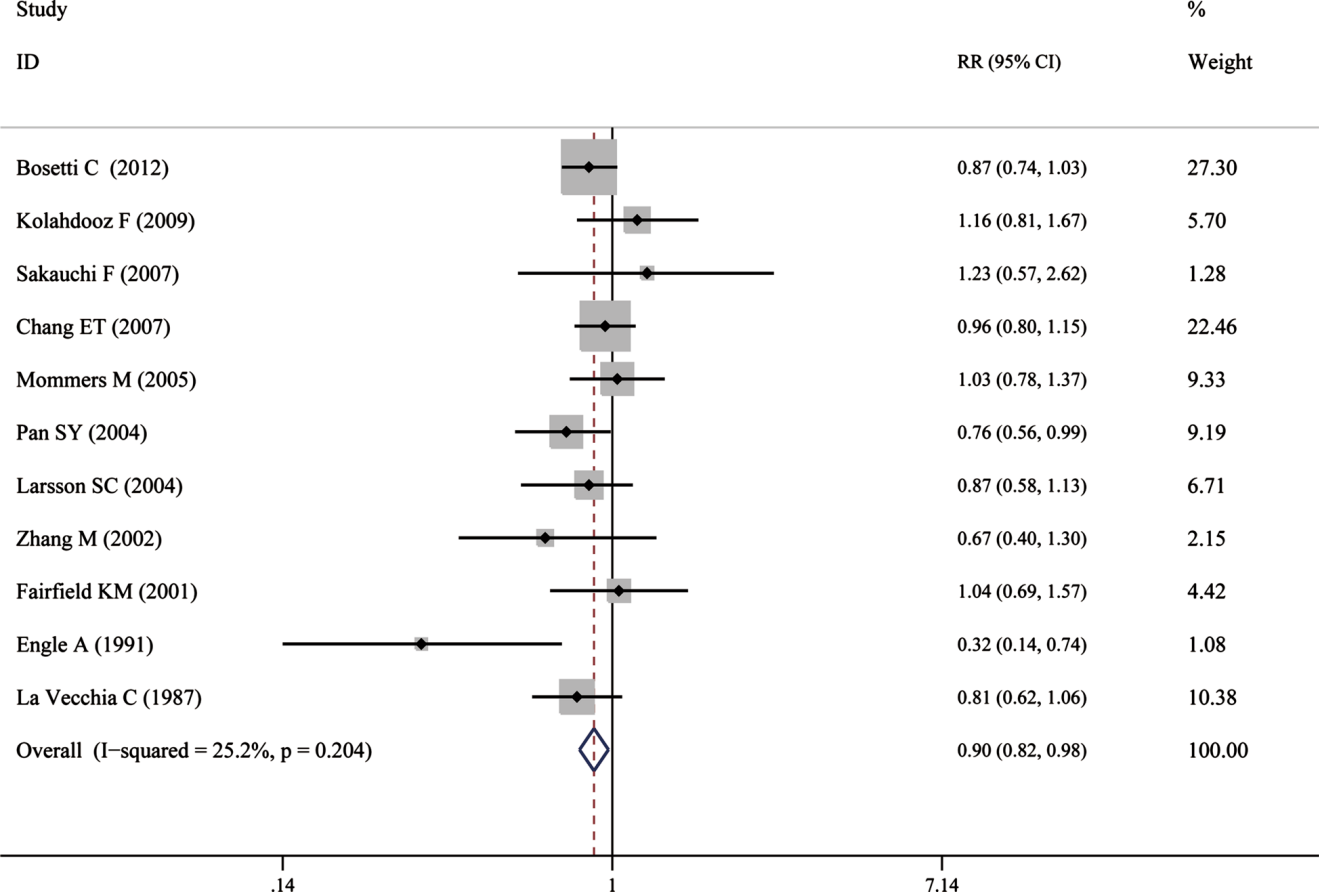
**Forest plot of the association between cruciferous vegetable consumption and risk of ovarian cancer.** The size of the shaded square is proportional to the percent weight of each study. The horizontal lines represent 95% CIs. The diamond data markers indicate the pooled ORs. A random-effect model was obtained.

The overall analysis of all 11 studies, including the case–control and cohort studies, found that cruciferous vegetable intake was associated with a reduced risk of ovarian cancer (n = 11, RR = 0.90; 95% CI: 0.82- 0.98, Figure 2). The subgroup analyses were conducted by the study designs, population or hospital based design and study sites. The effect of cruciferous vegetable consumption and ovarian cancer was detected discretely in the subgroup analyses. Cruciferous vegetable consumption was associated with a reduced risk of ovarian cancer in case–control studies (n = 6, RR = 0.84; 95% CI, 0.75-0.94) but not in cohort studies (n = 5, RR = 1.00; 95% CI, 0.85-1.11). When the data source was considered, the significant association was detected in the hospital-based studies (n = 4, RR, 0.82; 95% CI, 0.72-0.94) but not in the population-based group (n = 7, RR, 0.95; 95% CI, 0.85-1.07). When the geographical distribution was considered, only the studies conducted in the Europe demonstrated a significant result (n = 4, RR, 0.88; 95% CI, 0.79-0.99) (Table 2).

### Test for the heterogeneity

The heterogeneity was statistically significant when all the studies were pooled together (I^2^ = 25.2%; P = 0.284). When the included studies were excluded one by one and re-count the heterogeneity and the analyses did not identify change of the significance of the heterogeneity. The advanced subgroup analyses by study designs, study sites, and case group definitions demonstrated no significant results in the heterogeneity.

### Sensitivity analysis and publication bias

**Figure 3:**
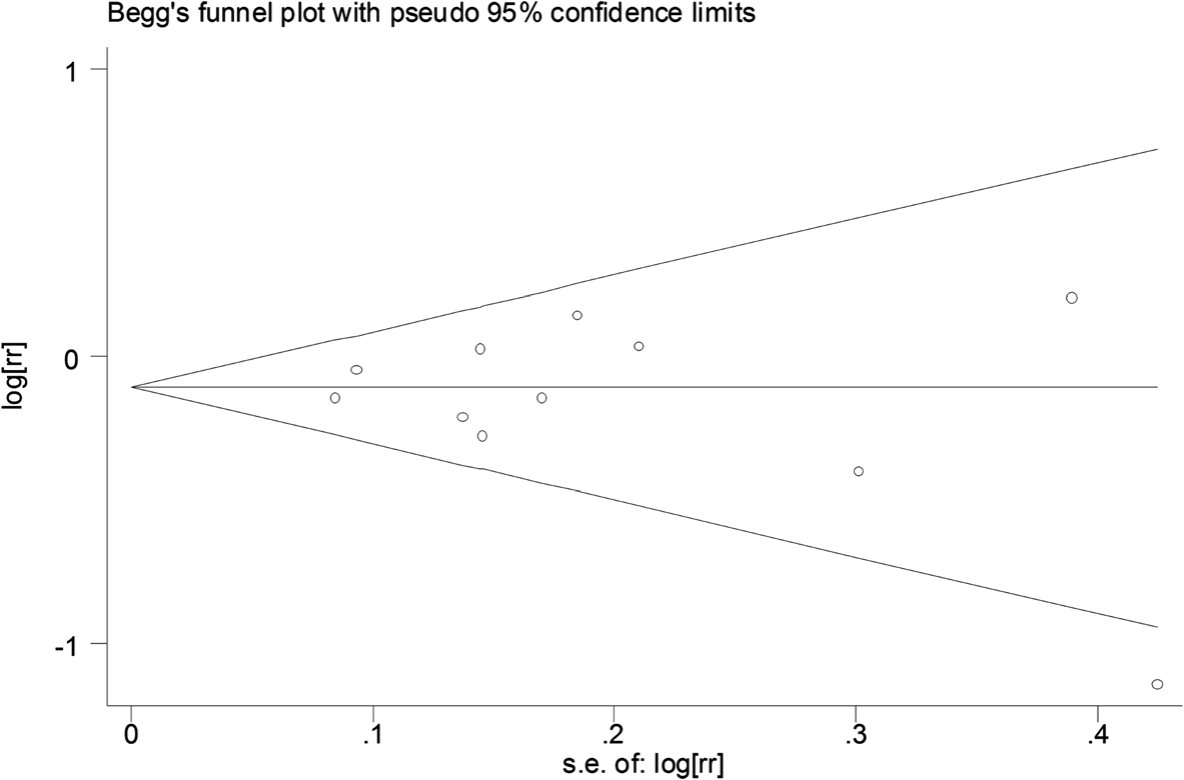
**Funnel plot of all the included studies.** Funnel plot of the RR (for the highest vs the lowest consumption categories) vs the standard error of the log RR for studies evaluating cruciferous vegetable consumption and ovarian cancer.

The sensitivity analysis was performed by excluding the included studies from the meta-analysis. The result showed that no one study could influence the significance of the conclusion. No indication of publication bias was observed in the literature on tea (Begg’s funnel plot, symmetrical, Figure 3; Begg’s test, *P* = 0.310; Egger’s test, *P* = 0.417).

## Discussion

A total of 4,306 cases in 375,562 controls in 11 independent studies were identified in this current meta-analysis. The result of this current meta-analysis, including 6 case–control and 5 cohort studies, indicated that cruciferous vegetable intake was associated with a reduced risk of ovarian cancer. When stratified by the study designs, although the meta-analysis from the case–control studies suggested a moderate reduction in risk, the results from the cohort studies were null. The heterogeneity was not significant when all the 11 studies were pooled in the meta-analysis. Publication bias was not detected in the meta-analysis. The results of the sensitivity analysis suggest that the conclusions of this study were quite robust.

Several in-vitro studies have tried to explore the link between cruciferous vegetables and cancer. Cruciferous vegetables are good sources of a variety of nutrients and phytochemicals that may have excellent cancer fighting properties [26]. The cancer-protective effects of cruciferous vegetables likely involve complex interactions of multiple mechanisms, and most research to date has focused on the capacity of cruciferous vegetable ingredients to alter biotransformation enzyme expression and activities. It has been long mentioned that cruciferous vegetables was associated with a reduced risk of ovarian cancer. In 1991, Engle et al. conducted a case–control study in which a total of 71 cases and 141 matched controls were investigated [21]. The result of this case–control studies demonstrated that cruciferous vegetable intake would reduce the incidence of ovarian cancer. In a population-based study in Canada, cruciferous vegetable consumption demonstrate prospective effect on the incidence of ovarian cancer [19]. In this meta-analysis, the results showed that cruciferous vegetable consumption was a prospective factor for the ovarian cancer.

In this study, the prospective effect of cruciferous vegetable consumption on the incidence of ovarian cancer was detected in case-controls but not cohort studies. Compared with retrospective studies, prospective studies are less susceptible to bias (e.g. recall bias, selection bias) due to their nature. Furthermore, case–control studies had a lower quality score than prospective studies. He difference between results from meta-analysis of case–control and cohort studies indicated that the association may have been changed by poor study methodologies. Likewise, in the subgroup analyses by type of control subjects, the protective effect in hospital-based control subjects was stronger than that in population-based ones, which might mean hospital-based case–control studies more inclined to selection bias. For the subgroup analysis of cruciferous vegetable intake and ovarian cancer risk by geographical site, the studies conducted in the Europe that egg consumption was a risk factor of the incidence of ovarian cancer but not the studies in the America, Asia and Australia. The geographical differences, the diet diversity and ethnic and genetic disparity are the possible reasons of the significant changes of the outcomes.

The strengths of this study include as follows: [1] we adopted a relative comprehensive literature search strategy in the acquisition of the potential included studies. We search the data base with key words of “diet” in combine with ovarian cancer and thus it would help to avoid missing includable articles. [2] All of the included studies included in this meta-analysis demonstrate a relative high quality. The results of the sensitivity analysis and the publication bias detection suggest that the conclusions of this study were quite robust, which may add strength to the conclusions drawn. [3] Consummate analyses, including detailed subgroup analyses, were conducted in this meta-analysis. The consummate analyses would provide us more detailed knowledge of the relation between egg consumption and risk of ovarian cancer.

As with any meta-analysis of observational studies, our study has several limitations. Firstly, half of the studies followed a case–control study design, and therefore there were recall and selection bias which are inherent to retrospective studies. Even through the subgroup analyses by the study designs were conducted, the efficiency was limited by the absence of enough cohort studies. Secondly, the data of included studies were not enough for us to conduct a dose–response meta-analysis. There points all indicate the requirement of additional well-designed studies in the future.

## Conclusions

In summary, this meta-analysis suggested that high intake of cruciferous vegetable can decrease risk of ovarian cancer. More in-depth studies are warranted to report more detailed results, including other specific vegetables within the cruciferous vegetable family, stratified results by ovarian cancer site, subtype of ovarian cancer, food preparation methods, or adjustment for potential confounders.

## Competing interests

The authors declare that they have no competing interests.

## Authors’ contributions

BH and TY conceived the study idea and designed the study. BH and XPL reviewed the literature and performed statistical analyses. BH, XPL and TY extracted data and drafted the manuscript. BH reviewed and edited the manuscript. All authors read and approved the final manuscript.
